# Autotrophic Production of the Sesquiterpene α-Humulene with *Cupriavidus necator* in a Controlled Bioreactor

**DOI:** 10.3390/bioengineering10101194

**Published:** 2023-10-14

**Authors:** Anne Sydow, Lucas Becker, Eric Lombard, Roland Ulber, Stephane E. Guillouet, Dirk Holtmann

**Affiliations:** 1 Industrial Biotechnology, DECHEMA Research Institute, Theodor Heuss Allee 25, 60486 Frankfurt, Germany; 2 Bioprocess Intensification, Institute of Bioprocess Engineering and Pharmaceutical Technology, Technische Hochschule Mittelhessen, Wiesenstrasse 14, 35390 Giessen, Germany; 3 TBI, Université de Toulouse, National Institute of Applied Sciences (INSA), 135 Avenue de Rangueil, 31077 Toulouse, France; 4 Institute of Bioprocess Engineering, University of Kaiserslautern-Landau, Gottlieb-Daimler-Straße 49, 67663 Kaiserslautern, Germany; 5 Institute of Process Engineering in Life Sciences, Karlsruhe Institute of Technology, Fritz-Haber-Weg 4, 76131 Karlsruhe, Germany

**Keywords:** CO_2_ conversion, *Cupriavidus necator*, autotroph, terpenes, α-humulene, in situ product removal

## Abstract

*Cupriavidus necator* is a facultative chemolithotrophic organism that grows under both heterotrophic and autotrophic conditions. It is becoming increasingly important due to its ability to convert CO_2_ into industrially valuable chemicals. To translate the potential of *C. necator* into technical applications, it is necessary to optimize and scale up production processes. A previous proof-of-principle study showed that *C. necator* can be used for the de novo production of the terpene α-humulene from CO_2_ up to concentrations of 11 mg L^−1^ in septum flasks. However, an increase in final product titer and space–time yield will be necessary to establish an economically viable industrial process. To ensure optimized growth and production conditions, the application of an improved process design in a gas bioreactor with the control of pH, dissolved oxygen and temperature including a controlled gas supply was investigated. In the controlled gas bioreactor, the concentration of α-humulene was improved by a factor of 6.6 and the space–time yield was improved by a factor of 13.2. These results represent an important step toward the autotrophic production of high-value chemicals from CO_2_. In addition, the in situ product removal of α-humulene was investigated and important indications of the critical logP value were obtained, which was in the range of 3.0–4.2.

## 1. Introduction

*Cupriavidus necator*, also known as *Ralstonia eutropha* or formerly *Alcaligenes eutrophus*, belongs to the β-proteobacteria and is a Gram-negative, rod-shaped bacterium with peritrichous flagella [1,2]. *C. necator* is a facultative chemolithotrophic organism that grows under both heterotrophic and autotrophic conditions [1]. In the absence of organic substrate, the organism is able to utilize CO_2_ as its sole carbon source by using the Calvin–Benson–Bassham cycle. This makes the use of *C. necator* economically and ecologically attractive, as CO_2_ is a cheap and abundant carbon source [3]. By combining water electrolysis with the ability to convert CO_2_, *C. necator* can also be used in bioelectrochemical synthesis, combining CO_2_ conversion with the sustainable generation of electrical energy to produce valuable chemicals [4,5,6]. The ability to convert a wide range of waste streams into these high-value products provides a great opportunity to develop a waste-based circular bioeconomy based on *C. necator* [7,8,9].

Under nutrient limitation (e.g., nitrogen limitation), the carbon is stored in *C. necator* as the biopolymer polyhydroxyalkanoates (PHA) such as polyhydroxybutyrate (PHB). Under chemolithotrophic conditions, *C. necator* can accumulate PHB as a carbon storage component up to 80% of cell dry weight [10]. PHB is a homopolymer and belongs to the PHAs. PHB has a crystalline structure that is described as stiff and brittle, but less thermostable material [11]. With the exception of biodegradability, PHB biopolymers are comparable to the petroleum-based polymers, polypropylene and polyethylene, and represent an environmentally friendly alternative [11]. In addition, metabolically engineered *C. necator* strains are capable of producing a wide range of industrially relevant products, e.g., terpenes (e.g., α-humulene, β-farnesene, lycopene [5,12,13], alkanes and alkenes [14,15], 3-hydroxypropionic acid [3], methyl ketones [16], iso-propanol [17] and iso-butanol [18]. Besides its broad substrate and product spectrum, *C. necator* is characterized by fast growth up to high cell densities, making it an attractive host for biotechnological applications. The facultative chemolithotrophy of *C. necator* facilitates genetic engineering compared to obligate chemolithotrophs. In recent years, more and more genetic tools have been developed [19,20,21,22]. The genetic tools for *C. necator* have been recently reviewed by Panich et al. and Pan et al. [23,24].

**Scheme 1:**
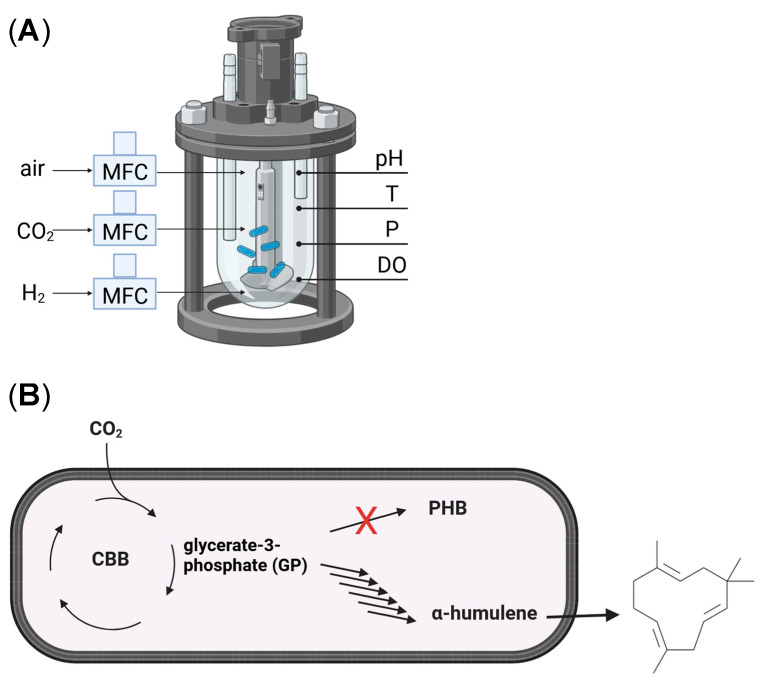
(**A**) Overview of the controlled bioreactor (MFC = mass flow controller, DO = dissolved oxygen, for a detailed description refer to the Materials and Methods section). (**B**) Schematic production of α-humulene forms CO_2_ in *C. necator* (CO_2_ is assimilated via the Calvin–Benson–Bassham (CBB) cycle, which yields 1 molecule glycerate-3-phosphate (GP) per 3 molecules CO_2_ fixed. Carbon can be stored in polyhydroxybutyrate (PHB) or used for α-humulene production starting from the same precursor acetoacetyl-CoA via the engineered mevalonate pathway (MVA) pathway or from glyceraldehyde-3-phosphate and pyruvate via the native methylerythritol 4-phosphate pathway (MEP). Here, a PHB-negative strain was used. The produced α-humulene was excreted in the media and afterward extracted by ISPR (in situ product removal).

In order to transfer the described potential of *C. necator* into technical applications, it is necessary to optimize and scale up the production processes. Therefore, the transfer from non-scalable septa flasks to controlled and scalable bioreactors was investigated. Several authors have investigated approaches for the production of PHB. Our aim is to optimize the production process for a higher-value product using the example of the terpene α-humulene. α-Humulene has anti-inflammatory [25,26] and antibacterial [27] properties. In addition, α-humulene is a precursor of the anticancer drug zerumbone, which means that the product is also considered to have anticancer activity [28,29]. In addition to its pharmaceutical properties, α-humulene is also found in plants like hops, giving them their characteristic smell and taste [30]. Therefore, α-humulene can be used in various applications. In the food and beverage industry, it can be used as a flavoring agent [31,32], while in medical research, its therapeutic [33] and pharmacokinetic [34] properties can be used. The starting point of our investigation was the ground-breaking work of Krieg et al. [12], who demonstrated for the first time chemolithoautotrophic de novo production of α-humulene from CO_2_. α-Humulene concentrations of up to 11 mg L^−1^ in autotrophic cultivation systems based on septa flasks were produced with 0.8 g L^−1^ biomass. However, an increase in final product concentration and space–time yield will be necessary to establish an economically viable industrial process. In order to improve the overall process, two strategies were investigated in detail. First, Krieg et al. used n-dodecane as the second phase for the required in situ product removal (ISPR) [12]. Here, different solvents were screened regarding their biocompatibility and potential as an extraction phase for α-humulene. The aim was to identify a more efficient, cost-effective and eco-friendly solvent. In addition, an improved process design was investigated. This included the implementation of the control of pH, dissolved oxygen, temperature and pressure, as well as a controlled gas supply. The reactor system and the production of α-humulene in *C. necator* are shown in the following schematic diagram (Scheme 1).

## 2. Materials and Methods

### 2.1. Plasmid and Strains 

The construction of the used pKR-hum plasmid was described previously [12]. The strain *C. necator* H16 PHB-4 (DSM-541) was purchased from Deutsche Stammsammlung für Mikroorganismen und Zellkulturen DSMZ (Braunschweig, Germany). 

### 2.2. Solvent Selection and Testing

In the literature, the recovery of extracellularly released α-humulene from culture broth has been performed by ISPR with n-dodecane [12,35]. In order to optimize the ISPR, several alternative solvents were tested. Therefore, different deep eutectic solvents (DES) and other promising solvents used in the literature for terpene extraction were compared with n-dodecane in terms of biocompatibility during cultivation with *C. necator* pKR-hum. Furthermore, the formation of a second liquid phase from the cultivation medium was a criterion to be tested in order to facilitate the removal of the α-humulene-containing solvent after the extraction process. Two DES systems, namely, tetrabutylammonium bromide (TBAB):1-octanol [36] and D-menthol:lauric acid [37], have been identified as potential alternatives to in situ product removal using n-dodecane, based on their applications in the literature and successful terpene extractions. Furthermore, the efficacy of acetophenone [38], methyl butyrate [39] and 1-cyclodextrin as extraction solvents was evaluated [40].

### 2.3. Preparation of Deep Eutectic Solvents 

D-menthol was used as hydrogen bond donor and lauric acid as hydrogen bond acceptor. They were heated together in a molar ratio of 2:1 for 2 h at 50 °C in a closed vessel until the solid components dissolved and a homogeneous liquid was formed [37]. TBAB was heated together with 1-octanol in a molar ratio of 1:2 for 2 h at 80 °C in a closed vessel until a homogeneous liquid was formed [36]. The deep eutectic solvents were then stored at room temperature until use and added to the fermentation broth after inoculation at 20% (*v*/*v*), similarly to the other extraction solvents tested.

### 2.4. Heterotrophic Cultivation for Solvent Selection

Lysogeny broth (LB) medium was used for the *C. necator* pKR-hum pre-cultures, which were inoculated from glycerol stock and cultivated at 180 rpm and 30 °C overnight (Infors HT Ecotron, Infors AG, Bottmingen, Switzerland). The media composition was 10 g L^−1^ NaCl, 10 g L^−1^ tryptone/peptone and 5 g L^−1^ yeast extract. Subsequently, the minimal medium (MM) for the main culture was prepared according to Table 1 and inoculated to a starting optical density (OD) of 0.1. Cultivation parameters were also 180 rpm and 30 °C (Infors HT Ecotron, Infors AG, Bottmingen, Switzerland) with 20 mL volume in 250 mL shake flasks. Trace elements were prepared as a stock solution in 0.05 M H_2_SO_4_ according to Table 1 and added to the minimal medium at 1:20,000. In addition, the media were supplemented with 15 µg mL^−1^ tetracycline hydrochloride for the recombinant *C. necator* pKR-hum strain. Cell growth was recorded by measuring the backscattered light from the suspension cells using the Cell Growth Quantifier (CGQ) from Scientific Bioprocessing (SBI), Pittsburgh, PA, USA. Data were recorded every 60 s over the entire cultivation period.

### 2.5. Media Compositions for the Seed Train of the Bioreactor Process

The rich medium consisted of 2.75% (*w*/*v*) dextrose-free tryptic soy broth (TSB, Becton Dickinson, Le Pont de Claix, France). Minimal medium A used for preculture in a flask was described in [14]. Fructose (20 g L^−1^) was used as the only carbon source. NH_4_Cl (0.5 g L^−1^) was used as nitrogen source to reach a biomass concentration of about 1 g L^−1^. Minimal medium B used for the cultures in the bioreactor consisted of 0.19 g L^−1^ nitrilotriacetic acid, 0.06 g L^−1^ ferrous ammonium citrate, 0.5 g L^−1^ MgSO_4_·7H_2_O, 0.01 g L^−1^ CaCl_2_·2H_2_O, and 1 mL of trace element solution. The trace element solution composition was 0.3 g L^−1^ H_3_BO_3_, 0.2 g L^−1^ CoCl_2_·6H_2_O, 0.1 g L^−1^ ZnSO_4_·7 H_2_O, 0.03 g L^−1^ MnCl_2_·4H_2_O, 0.03 g L^−1^ Na_2_MoO_4_·2H_2_O, 0.02 g L^−1^ NiCl_2_·6H_2_O, 0.01 g L^−1^ CuSO_4_·5H_2_O. (NH_4_)_2_SO_4_ (2 g L^−1^) was used as a nitrogen source to reach a biomass concentration of about 4 g L^−1^. After autoclaving the above medium, 40 mL of a sterile phosphate solution containing 224 g L^−1^ of Na_2_HPO_4_·12H_2_O and 37.5 g L^−1^ of KH_2_PO_4_ was aseptically added to the bioreactor.

### 2.6. Cultivation Conditions in the Seed Train of the Bioreactor Process

After thawing at room temperature, one glycerol stock was streaked on a TSB agar Petri dish. The plate was incubated for 36 h at 30 °C. One isolated colony was used to inoculate the first seed culture grown for 24 h in 5 mL TSB supplemented with 10 mg L^−1^ gentamycin and 200 mg L^−1^ kanamycin, in a 50 mL baffled Erlenmeyer flask. This culture was used to inoculate a 250 mL Erlenmeyer baffled flask containing 50 mL of mineral medium A. The seed was cultivated at 30 °C and 100 rpm for 18 h. The culture was then centrifuged (5 min, 10,000× *g*), the supernatant was discarded and the cell pellet was resuspended in 30 mL sterile fresh medium B to eliminate the residual fructose. The suspension was then used to inoculate the bioreactor containing 300 mL of sterile mineral medium B.

### 2.7. Autotrophic Culture Conditions and Bioreactor System

The cultivations were performed in a 500 mL total volume Xplorer^®^ (https://www.xplorersoftware.com/, accessed on 1 September 2023) bioreactor (HEL Ltd., Borehamwood, UK) modified as described in [41]. The liquid working volume was 330 mL. The bioreactor was fed with individual gases through three spargers in order to deliver air, pure CO_2_ and pure H_2_ independently. A fourth inlet gas was added in the headspace for pure nitrogen supply as a safety system. Each gas inlet was controlled by a gas mass flow meter. The bioreactor was equipped with pH, dissolved oxygen (DO), temperature and pressure controllers. The Xplorer^®^ software handled the online monitoring and control systems of the reactor. Both CO_2_ and O_2_ in the outlet gases were analyzed using a Tandem Pro gas analyzer (Magellan Instruments, Borehamwood UK). The bioreactor was safely controlled by the oxygen concentration in the headspace in order to avoid any explosive risk. The headspace was flushed with pure nitrogen if the O_2_ surpassed 5% in the headspace. The DO level in the reactor was controlled below 5% of air saturation at atmospheric pressure by varying stirring speed and/or inlet air flow rate and overpressure up to 5 bars. Stirring speed was kept at 795 +/− 15 rpm by magnetic stirring and temperature was maintained at 30 °C using the poly-BLOCK (HEL Ltd., UK). For pH control, 5 M (9.5%) ammonium hydroxide and 42.5% phosphoric acid were provided as base and acid, respectively. Furthermore, ammonium hydroxide could serve as additional nitrogen source. As the amount of feed solution added to the reactor and sample volume taken from the reactor during cultivation was in the same range (approx. 25 mL), no volume correction was performed. α-Humulene production was induced by adding 11 mM L-rhamnose at 18 h. At the same time, n-dodecane (20% *v*/*v*) was added by a syringe to the culture to extract *α*-humulene. Samples were taken through a septum.

### 2.8. Characterization of the Fermentation Process

The cell growth was followed by spectrophotometric measurements at 600 nm with a DR3900 spectrophotometer (Hach, Loveland, CO, USA) after a calibration against cell dry weight measurements to evaluate cell growth. For cell dry weight determination, the culture medium was harvested and filtrated on 0.2 μm pore-size polyamide membranes (Sartorius AG, Göttingen, Germany), which were then dried to a constant weight at 60 °C under partial vacuum (200 mmHg, i.e., approximately 26.7 kPa). Cell viability was measured with a BD Accuri C6^®^ flow cytometer (BD Biosciences, Franklin Lakes, NJ, USA) after cell staining with propidium iodide (PI) dye (Molecular Probes, Invitrogen, Waltham, MA, USA) commonly used to monitor membrane integrity as an indicator for viability.

### 2.9. Quantification of α-Humulene with GC-MS

To measure the α-humulene content in the solvents, 100 µL of the centrifuged extractant supernatant (5 min, 1000× *g*) was taken in GC glass vials. Subsequently, the vials were stored at –20 °C and 900 µL of acetone was added shortly before the GC-MS measurement. To prepare the calibration standards, α-humulene was dissolved in acetone from a stock solution (PhytoLab, Vestenbergsgreuth, Germany). Samples were measured in GC-MS (7890B GC-MS system with 5977B GC/MSD, Agilent Technologies, Santa Clara, CA, USA) with detection at airflow 450 mL min^−1^, N_2_ flow 45 mL min^−1^ and FID 250 °C. The oven temperature profile according to Table 2 was set with a heating step at 16 °C min^−1^. Measurements were performed with 100% acetonitrile as rinse solvent and a sample injection volume of 1 µL using an HP-5ms column (Agilent 19091S-433—30 m × 250 µm × 0.25 µm, Agilent Technologies, Santa Clara, CA, USA). Analysis was performed using the single ion monitoring method (SIM) at 204.2 *m*/*z* for α-humulene and 218.4 *m*/*z* for zerumbone when used as an internal standard.

### 2.10. Statistical Analyses

Concentrations of metabolites and biomass are given as the mean value of 2 to 3 independent analyses. The error on the specific growth rate (determined as being the slope of ln [OD] = f(t)) was calculated as the standard deviation of the slope. The instantaneous specific growth and α-humulene production rates were calculated upon the fitting process of the experimental data and the derivative calculation.

## 3. Results

### 3.1. Identification of the Most Promising Solvent for In Situ Product Removal

**Figure 1:**
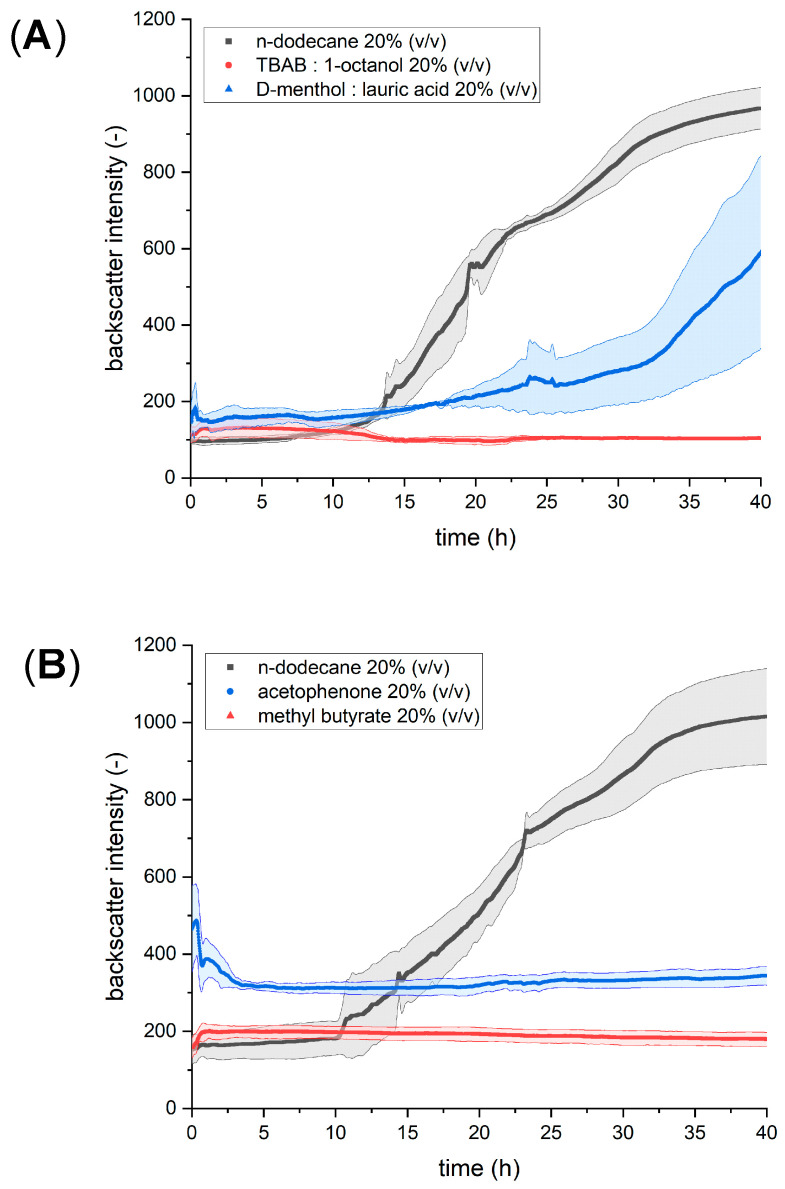
(**A**) Heterotrophic growth of *C. necator* pKR-hum in minimal media under the influence of different deep eutectic solvents for in situ product removal, with standard error (n = 3), (**B**) Heterotrophic growth of *C. necator* pKR-hum in minimal media under the influence of different solvents for in situ product removal, with standard error (n = 3).

As described above, various solvents were tested and their suitability as alternatives to n-dodecane was investigated. In the first step, the influence on the growth of *C. necator* was investigated. The heterotrophic growth of *C. necator* pKR-hum in a minimal medium was reduced by the addition of the deep eutectic solvents compared to n-dodecane (Figure 1A). The addition of 20% (*v*/*v*) of 1:2 molar TBAB:1-octanol resulted in no growth after inoculation. The addition of 20% (*v*/*v*) of the 2:1 molar D-menthol:lauric acid mixture resulted in reduced growth with a prolonged lag phase up to 20 h and a reduced final cell concentration of 300–800 backscatter intensity after 40 h compared to 900–1050 backscatter intensity with n-dodecane. Nevertheless, successful separation and formation of a second phase above the minimal medium was observed for both deep eutectic solvents identical to n-dodecane.

When 20% (*v*/*v*) of the alternative solvents acetophenone and methyl butyrate were added to the cultivation process, no growth was detected after inoculation compared to the standard extraction solvent n-dodecane (Figure 1B). Both alternative solvents successfully formed a second phase that separated from the minimal medium, with 20% (*v*/*v*) methyl butyrate forming an upper phase identical to 20% (*v*/*v*) n-dodecane and 20% (*v*/*v*) acetophenone forming a phase that appeared at the bottom of the shake flask. In addition, a strong unpleasant odor was detected when acetophenone and methyl butyrate were used as solvents compared to the menthol-containing deep eutectic solvent or n-dodecane. Replacement of n-dodecane with 1-cyclodextrin in the experimental setup was rejected because the addition of the soluble 1-cyclodextrin to the minimal medium did not form an additional second phase, which was used as a decision criterion. The non-biocompatible solvents (TBAB:1-octanol, acetophenone and methyl butyrate) must therefore have an inhibitory effect on the growth of *C. necator*.

The biocompatibility of solvents can be assessed using logP values. The logP is the logarithmic partition coefficient of a target compound in a biphasic system consisting of octanol and water and is a measure of the hydrophobicity of a molecule [42,43]. The lower the logP, the greater its hydrophilicity and thus its tendency to accumulate in the aqueous phase, where microorganisms reside, and interfere with the physiological processes of the cells [44]. The critical logP, which prevents further metabolic activity, is strongly dependent on the microorganism: e.g., for *E. coli,* the critical logP is reported to be 3.4 [45], whereas *S. cerevisiae* has a critical logP between 4.0 [46] and 5.6 [47]. When comparing the logP values, it is noticeable that n-dodecane (6.1), D-menthol (3.0) and lauric acid (4.2) have much higher values than 1-octanol (3.0), acetophenone (1.6) and methyl butyrate (1.3). Therefore, it can be assumed that the critical logP of *C. necator* is between 3.0 and 4.2.

**Figure 2:**
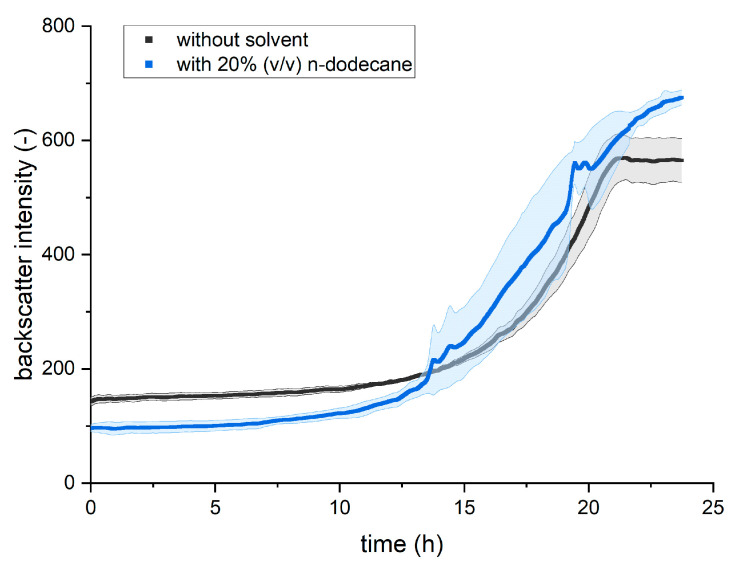
Heterotrophic growth of *C. necator* pKR-hum in minimal media under the influence of 20% (*v*/*v*) n-dodecane and without solvent, with standard error (n = 3).

The heterotrophic growth behavior in Figure 2 shows a similar pattern between the bacterial growth without solvent and the addition of 20% (*v*/*v*) n-dodecane, considering the standard deviation. In both experiments, the length of the lag phase is about 10 h and the cultures in exponential phases behave similarly. The experiment with 20% (*v*/*v*) n-dodecane reaches an increased final backscatter value at 25 h. However, this increased backscatter intensity value can be attributed to the additional n-dodecane, since an offline optical density measurement at 600 nm for both experiments showed a value of 3.0 and 3.1, respectively, at the end of the cultivation. Therefore, the slightly increased backscatter value can be explained by the addition of phase-forming solvents in general.

### 3.2. Optimization of the Autotrophic α-Humulene in a Controlled Gas Bioreactor

**Figure 3:**
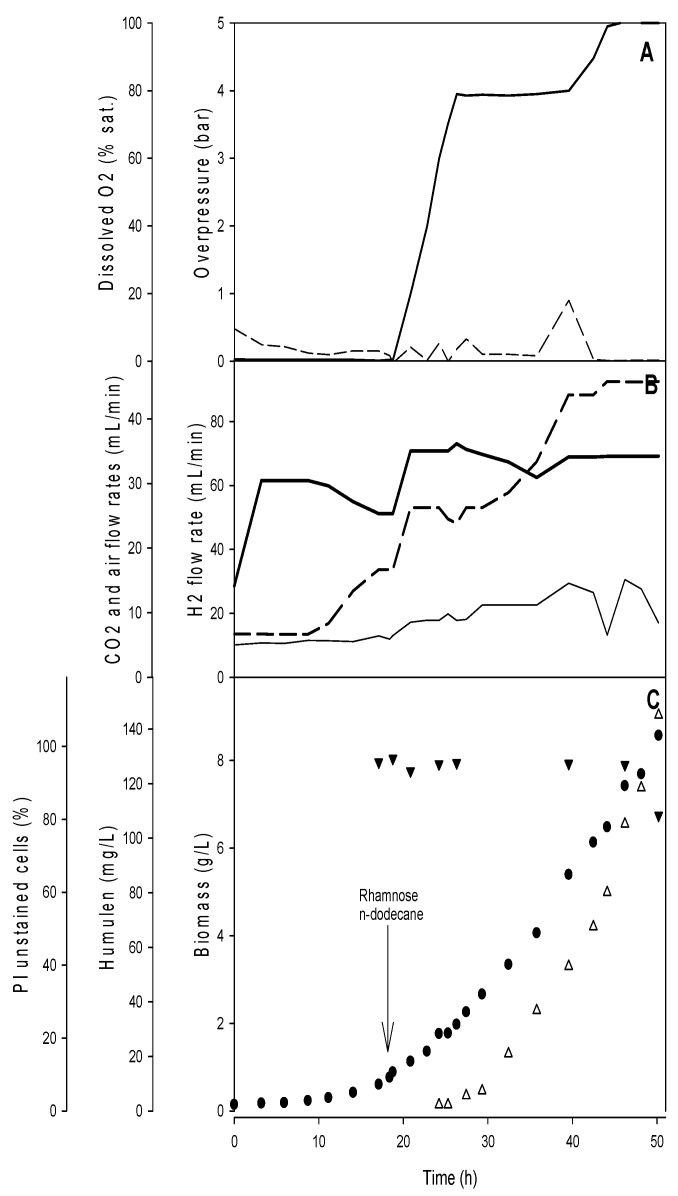
Autotrophic growth of *C. necator* and α-humulene production in the gas bioreactor: (**A**) Time course of overpressure (thick line) and partial pressure of dissolved oxygen (dashed line). (**B**) Evolution of the gas flow rates of H_2_ (solid thick line), Air (dashed line) and CO_2_ (solid thin line). (**C**) Evolution of biomass (●), α-humulene concentrations (∆) and percentage of PI unstained cells (▼).

Autotrophic α-humulene production was investigated in a dedicated gas bioreactor, as described in [41] and using the above-mentioned *C. necator* strain [12]. The cultivation was initiated by sparging the three independent gases (H_2_, air and CO_2_) in the reactor. The initial condition was set at 77% H_2_, 4% O_2_ and 5% CO_2_ gas mixture in the reactor, allowing a H_2_-rich and O_2_-low environment to be reached. The level of dissolved oxygen was monitored throughout the cultivation by increasing the ratio of air/H_2_ flow rate and the overpressure in the gas bioreactor in order to react to the increasing microbial oxygen demand and to keep the headspace O_2_ level below 5% (Figure 3). During the cultivation process, the gas flow rates ranged from 30 to 70 mL min^−1^ for H_2_, 6.7 to 45 mL min^−1^ for air and 2 to 8 mL min^−1^ for CO_2_. The *α*-humulene production strain *C. necator* (pKR-hum) was grown in the bioreactor for 50 h, the highest specific growth rates were obtained during the ten hours before induction at 18 h with µ = 0.13 ± 0.01 h^−1^. Two hours after induction, the growth rate decreased. Besides the heterologous gene expression, which reduced the specific growth rate after induction, limitations in oxygen transfer occurred and limited the process performance. The dissolved oxygen concentration was adjusted to between 1 and 3%, but starting from 42 h after inoculation the value was constantly zero. The portion of the oxygen in the inlet gas flow could not be further increased because the lower explosion limit was reached (exhaust gas with 4% oxygen in a hydrogen-dominant gas composition). Furthermore, the reactor pressure limit of six bars was reached and the total gas flow was already high with 120 mL min^−1^. As oxygen concentration had no impact on heterotrophic *α*-humulene production, whereas cell growth was identified as the most important parameter for the production of the target compound, the autotrophic process was continued. The absence of growth inhibition by the presence of α-humulene (up to 1 g L^−1^) or n-dodecane (up to 20%) was previously shown [12]. The final biomass concentration reached after 50 h was 8.5 g_CDW_ L^−1^. α-Humulene was detected after 24 h at 2 mg L^−1^ and continued to reach a final concentration of 146 mg L^−1^ at the end of the fermentation leading to an overall productivity of 4.6 mg L^−1^ h^−1^. Consumption of the inducer L-rhamnose was excluded (data not shown). A slight decrease of approx. 5% can be explained by the dilution due to base addition for the pH control. The specific *α*-humulene production rate continuously increased during cultivation to the highest value of 1.8 mg g_CDW_^−1^ h^−1^ reached at the end of cultivation, corresponding to a specific production of 16 mg g_CDW_^−1^. The cell’s viability was assessed by flow cytometry during the cultivation after staining the cells with propidium iodide (PI). The percentage of PI-unstained cells (viable cells), remained high at over 95% throughout the fermentation, even after the reactor was pressurized. Only at the endpoint, the viability dropped to 81% of PI-unstained cells.

## 4. Discussion and Outlook

In terms of the solvent evaluation, it could be shown that the addition of 20% (*v*/*v*) n-dodecane for in situ product removal of α-humulene does not reduce bacterial growth compared to its absence and is, therefore, a suitable biocompatible solvent. The alternative solvents were all found to be non-biocompatible at the 20% (*v*/*v*) concentration tested, as no bacterial growth occurred upon their addition. Only the deep eutectic solvent tested, consisting of D-menthol:lauric acid 20% (*v*/*v*), showed limited biocompatibility with a longer lag phase and lower final cell concentration. Therefore, n-dodecane can still be considered as the currently most promising solvent for ISPR in *C. necator*-based production processes. It is therefore advisable to perform the testing of similar alkanes in the next step. Table 3 shows a comparison of the process parameters resulting from previously published cultivations in septum flasks and the control gas bioreactor presented here. Using the controlled gas bioreactor, biomass concentration was improved by a factor of 3.2 and product concentration by a factor of 6.6. While the biomass-specific productivity in the controlled bioreactor (mg g_CDW_^−1^) was improved by a factor of 2, the space–time yield was even further increased by a factor of 13.2. Thus, the promising results from the cultivations in simple septa flasks could be significantly improved by using a controlled bioreactor, as intended. Furthermore, it was shown that a safe cultivation process can be carried out without safety risks using oxyhydrogen (Knallgas) gas and *C. necator*, which is in agreement with the literature [10,41,48] and shows the high potential of also using *C. necator* in technical reactors. Milker et al. showed the production of α-humulene up to 2 g L^−1^ using the same strain in a fed-batch mode with fructose as the carbon source [35]. Although this final concentration is 13.7 times higher than in the autotrophic system shown here, the productivity of the processes is in a comparable range. The productivities of the heterotrophic and autotrophic processes were 5.52 and 4.63 mg L^−1^ h^−1^, respectively.

The next step is to further optimize production. This can be achieved by improving the production load, for example, by optimizing the MVA path. There is also further potential on the engineering side, where media optimization or a detailed study of the factors influencing product formation in the bioreactor could be helpful.

## 5. Conclusions

By using a gas bioreactor with the control of pH, dissolved oxygen and temperature, as well as a controlled supply of the required gaseous substrates CO_2_, H_2_ and O_2_, the microbial α-humulene production with C. necator was drastically improved Although the final product concentrations are still significantly higher in heterotrophic production with the strain, this shows the enormous potential of autotrophic production with *C. neactor*.

## Figures and Tables

**Table 1:** Composition of the minimal medium used (MM).

Medium Component MM	Concentration (g L^−1^)	Trace Element Stock	Concentration (g L^−1^)
Na_2_HPO_4_	2.895	FeSO_4_ · 7 H_2_O	15.0
NaH_2_PO_4_ · H_2_O	2.707	MnSO_4_ · H_2_O	2.4
CaSO_4_ · 2 H_2_O	0.097	ZnSO_4_ · 7 H_2_O	2.4
K_2_SO_4_	0.170	Na_2_MoO_4_ · 2 H_2_O	1.8
(NH_4_)_2_SO_4_	0.943	CuSO_4_ · 5 H_2_O	0.48
MgSO_4_ · 7 H_2_O	0.8	NiSO_4_ · 6 H_2_O	1.5
D-Fructose	4.0	CoSO_4_ · 7 H_2_O	0.04
Trace elements	1:20,000 from stock		

**Table 2:** Oven temperature profile for quantification of α-humulene via GC-MS.

Temperature (°C)	Time (min)
70	0
70	1.5
200	9.625
200	10.125

**Table 3:** Comparison of incubation and production parameters for autotrophic *α*-humulene production by *C. necator* (pKR-hum).

	Septum Flask, n = 3 [12]	Controlled Gas Bioreactor [This Work]
Substrate feed	(Fed)-batch	Fed-batch
Externally provided gases	H_2_, CO_2_, O_2_	H_2_, CO_2_, Air
Minimal medium at t = 0 (mL)	20	300
n-Dodecane (*v*/*v*)	20%	20%
n-Dodecane added	At the beginning of cultivation	At the induction time point
Inducer (mM)	11	11
Mixing type	Incubating shaker	Magnetic stirring
Mixing frequency (rpm)	180	795 ± 15
pH control	No	Yes (≥6.6)
CDW (g L^−1^)	2.69 ± 0.05	8.57
µ (h^−1^)	0.12 ± 0.00	0.13
α-humulene titer (mg L^−1^)	22.0 ± 2.2	146
Space-time yield (mg L^−1^ h^−1^)	0.35 ± 0.02	4.63
Specific productivity (mg g_CDW_^−1^)	8.62 ± 1.13	17.1

## Data Availability

The data presented in this study are available on request from the corresponding author.
